# Benchmarking of methods that identify alternative polyadenylation events in single-/multiple-polyadenylation site genes

**DOI:** 10.1093/nargab/lqaf056

**Published:** 2025-05-14

**Authors:** Qiuxiang Tian, Quan Zou, Linpei Jia

**Affiliations:** College of Information Science and Engineering, Hunan University, Changsha, Hunan, 410082, China; School of Information Technology and Administration, Hunan University of Finance and Economics, Changsha, 410205, China; Yangtze Delta Region Institute (Quzhou), University of Electronic Science and Technology of China, Quzhou, Zhejiang, 324000, China; Department of Nephrology, Xuanwu Hospital, Capital Medical University, No. 45 Changchun Street, Beijing, 100053, China

## Abstract

Alternative polyadenylation (APA) is a widespread post-transcriptional mechanism that diversifies gene expression by generating messenger RNA isoforms with varying 3′ untranslated regions. Accurate identification and quantification of transcriptome-wide polyadenylation site (PAS) usage are essential for understanding APA-mediated gene regulation and its biological implications. In this review, we first review the landscape of computational tools developed to identify APA events from RNA sequencing (RNA-seq) data. We then benchmarked five PAS prediction tools and seven APA detection algorithms using five RNA-seq datasets derived from clear cell renal cell carcinoma (ccRCC) and adjacent normal tissues. By evaluating tool performance across genes with either single or multiple PASs, we revealed substantial variation in accuracy, sensitivity, and consistency among the tools. Based on this comparative analysis, we offer practical guidelines for tool selection and propose considerations for improving APA detection accuracy. Additionally, our analysis identified CCNL2 as a candidate gene exhibiting significant APA regulation in ccRCC, highlighting its potential as a disease-associated biomarker.

## Introduction

Alternative polyadenylation (APA) is a pervasive mechanism that enables a single gene to generate multiple transcript isoforms by selecting different cleavage and polyadenylation sites (PASs). This process modulates the 3′ end of messenger RNAs (mRNAs) and profoundly influences RNA stability, localization, translation, and interaction with regulatory factors. RNA polymerase II transcription is coupled with key mRNA processing steps, including 5′ capping, splicing, and cleavage and polyadenylation at the 3′ ends of pre-mRNAs [1].

**Figure 1.**
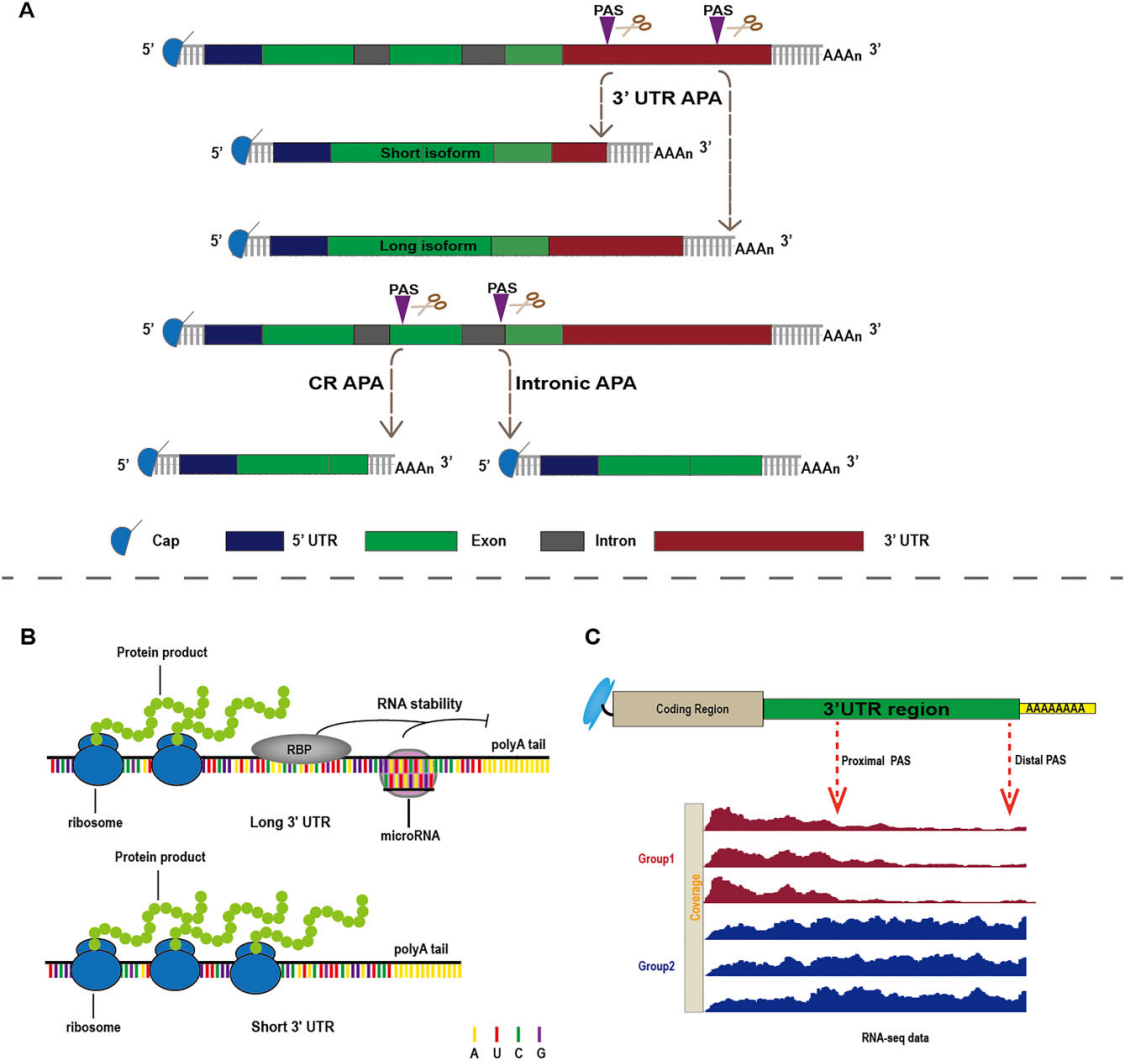
Representation categories and functions of APA and the analysis of APA. (**A**) 3′ UTR APA containing two PASs in the 3′ UTR, producing short and long mRNA isoforms. IPA occurring in the introns and CR APA in exon generating truncated coding region and 3′ UTR-lacking mRNA isoform. The semicircle denotes the 5′ cap; the rectangle adjacent to it represents the 5′ UTR; subsequent rectangles correspond to exons; and the final rectangle indicates the 3′ UTR. (**B**) mRNA isoforms with longer 3′ UTRs typically contain more miRNA and RBP binding sites, which can affect the stability of these mRNA isoforms, whereas shorter UTR mRNA isoforms often lead to the production of more protein. (**C**) The upper diagram illustrates the gene structure, while the lower diagram shows how APA software detects PASs by identifying change points in sequence abundance within the UTR region.

Most eukaryotic genes harbor multiple PASs [2], enabling distinct APA patterns, such as tandem-APA, which generates mRNAs with variable untranslated region (UTR) lengths; intronic-APA (IPA), which utilizes cryptic PASs in introns; and **coding region APA (CR APA)**, which can lead to truncated coding sequences (CDSs) [3]. These APA types contribute to transcriptome and proteome diversity (Fig. 1A) [4, 5]. In fact, APA affects over 70% of human protein-coding genes [6], underscoring its ubiquity and functional relevance.

The regulatory impact of APA is particularly evident in 3′ UTR dynamics. Shortened 3′ UTRs often escape regulation by RNA-binding protein (RBP) and microRNA (miRNA) target sites, resulting in enhanced mRNA stability and translational output—features that can promote oncogene expression (Fig. 1B) [7, 8]. In contrast, longer 3′ UTRs tend to harbor more regulatory elements, potentially leading to mRNA destabilization or translational repression [9]. Intriguingly, some studies have reported that longer 3′ UTR isoforms may paradoxically have shorter half-lives, emphasizing the nuanced regulatory roles of APA [10].

Given these complex biological consequences, precise and transcriptome-wide quantification of PAS usage is essential for dissecting APA mechanisms across cell types, developmental stages, and disease states. High-throughput RNA sequencing (RNA-seq) has become an indispensable tool for this purpose [11]. To enrich 3′ ends, specialized RNA-seq protocols are employed and generally fall into two methodological categories: RNA manipulation-based protocols, which which retain strand specificity and achieve high PAS resolution [12–17]; and oligo (dT) priming-based protocols, which are more scalable and suitable for bulk sample processing [6, 18, 19–24]. These techniques have revealed widespread, tissue-specific APA regulation and uncovered thousands of previously unannotated PASs.

Nonetheless, due to the limited availability of 3′ end-enriched datasets, standard RNA-seq remains the most commonly used modality for APA analysis. To extract APA-related features from such data, a diverse array of computational tools has been developed (Fig. 1C) [25]. These methods employ varying strategies, including read density (RD) modeling, change-point detection, and machine learning-based classification [1, 5]. However, the lack of standardized annotations, performance metrics, and benchmark datasets complicates the interpretation and comparison of results across in different tools. Prior benchmark studies have reported low concordance of PAS detection among existing methods, likely due to differences in datasets, parameter settings, and/or performance metrics [26–28], highlighting the need for rigorous and context-aware benchmarking.

To address these limitations, we performed a systematic benchmarking study using RNA-seq data from clear cell renal cell carcinoma (ccRCC) and adjacent normal tissues. A distinctive feature of our analysis is the stratification of genes based on PAS complexity, enabling a detailed performance evaluation across single- and multi-PAS genes. By quantifying the sensitivity, precision, and agreement of tools under different gene architectures, our study provides insights into their relative strengths and limitations. These findings serve as a practical guide for researchers seeking to select APA tools tailored to their specific datasets and research objectives.

## Current methods for predicting PAS using RNA-seq data

Numerous studies on post-transcriptional regulation have underscored the significance of APA in shaping 3′ UTR and architecture and modulating poly (A) tail length. These APA-mediated variations significantly influence transcript stability and gene expression, making the accurate detection of poly (A) signals essential for elucidating dynamic APA regulation within 3′ UTRs.

In our comprehensive overview, we systematically categorized the existing bioinformatics tools for PAS identification and APA dynamics quantification from RNA-seq data into two major groups: (i) methods relying on priori PAS annotations, and (ii) methods that infer PASs based on 3′ end alignment patterns in mRNA sequencing data. Using this classification framework, we selected 21 representative tools, evaluating their capabilities in PAS identification, absolute and relative PAS quantification, differential PAS usage analysis, and APA event detection (Table 1 and Supplementary Table S1). From these, we selected nine tools developed in the past 5 years (given in bold in Table 1) for in-depth benchmarking of their performance in PAS and APA event detection.

**Table 1.** Methods applied to detect PAS

Name	Year	Key features	PAS identification	Absolute PAS quantification	Relative PAS quantification	Differential PAS usage	Reference
Dapars	2014	Read density	Yes	No	Yes	Yes	[41]
ChangePoint	2014	Change-point mode Read density	No	No	No	No	[39]
GETUTR	2015	Read coverage	Yes	No	Yes	Yes	[38]
IsoSCM	2015	Read density Bayesian model	Yes	No	Yes	Yes	[40]
Roar	2016	Read density	No	Yes	Yes	Yes	[29]
QAPA	2018	Annotated poly (A) sites Relative usage	No	Yes	Yes	No	[30]
PAQR_KAPAC	2018	Annotated poly (A) sites Read coverage	Yes	No	Yes	No	[31]
APAtrap	2018	Sliding window strategy Mean squared error model	Yes	Yes	Yes	Yes	[43]
IntMap	2018	Constrained probabilistic model Read alignments	No	No	No	No	[65]
MountainClimber	2019	Read density	Yes	Yes	Yes	Yes	[46]
CSI-UTR	2019	Read alignments Gene-based analyses	No	No	No	Yes	[32]
DeeReCT-APA	2022	Deep learning method CNN–LSTM	Yes	Yes	No	No	[66]
**APAlyzer**	2020	Annotated poly (A) sites Read density	No	No	Yes	Yes	[33]
**Dapars2**	2021	Read density	Yes	No	Yes	No	[42]
**APA-Scan**	2022	Read density	No	No	Yes	Yes	[34]
**flexiMAP**	2021	Read density	No	No	No	Yes	[35]
**diffUTR**	2021	Read density	No	No	No	Yes	[36]
**TAPAS**	2021	Read density	Yes	No	No	Yes	[45]
**REPAC**	2023	Isometric log ratio Differential polyadenylation site usage	Yes	Yes	No	Yes	[47]
**APAIQ**	2023	Deep learning model	Yes	No	No	No	[44]
**PolyAMiner-Bulk**	2024	Deep learning algorithm	Yes	Yes	No	No	[48]

### Methods relying on a priori annotations of PAS

Identifying PAS from RNA-seq data based on known PAS annotations is among the simplest and most computationally efficient strategies. However, a key limitation of this approach is its inability to discover novel PAS sites. The first category of tools relies on pre-annotated PASs and includes methods, such as [29] QAPA [30], PAQR_KAPAC [31], CSI-UTR [32], APAlyzer [33], APA-Scan [34], flexiMAP [35], and diffUTR [36].

APAlyzer is a bioinformatics package designed to analyze 3′ UTR APA, IPA, and differential gene expression from RNA-seq data using annotated PASs in from the PolyA_DB database. For genes with multiple PAS in their 3′ UTRs, APAlyzer segments the region into a constitutive UTR, spanning from the stop codon and the first PAS, and an alternative UTR, spanning from the first and the last PAS. RDs are calculated for both regions. For 3′ UTR APA analysis, the PASEXP_3UTR function focuses on the first and last PASs located in the last exon of each gene. The relative APA usage between two conditions is quantified by relative expression (RE) difference, and statistical significance is assessed via the APAdiff function, which supports multiple statistical testing methods depending on the experimental design.

APA-Scan supports PAS identification using either predicted or experimentally validated polyadenylation signals. It estimates the abundance of long and short 3′ UTR isoforms from RNA-seq data. In its default mode, APA-Scan defines the 3′ UTR based on the end of the longest annotated transcript of each gene. Peaks identified in the 3′ end sequencing data are treated as potential cleavage sites. In the absence of such data, APA-Scan detects canonical PAS motifs (typically two variants of hexamers: AATAAA and ATTAAA) within the 3′ UTR, which are referred to as APA-Scan^PAS^.

diffUTR is a Bioconductor package that enhances differential exon usage (DEU) analyses for detecting differential 3′ UTR usage. It integrates existing DEU frameworks with curated APA site databases. The tool annotates noncoding bins from protein-coding transcripts as UTR and the remaining bins as CDS. These regions are then binarized and quantified using the *Rsubread* package [37], followed by DEU analysis to detect significant differences in UTR usage between conditions.

### Methods relying on 3′ end alignment information of mRNA transcripts

The second category of APA detection tools comprises methods that infer PASs based on read alignment patterns rather than relying on prior PAS annotations. Tools in this group include GETUTR [38], ChangePoint [39], IsoSCM [40], DaPars [41], DaPars2 [42], APAtrap [43], APAIQ [44], TAPAS [45], MountainClimber [46], REPAC [47], and PolyAMiner-Bulk [48]. These methods typically analyze RNA-seq read coverage across the 3′ UTR to identify abrupt fluctuations in RD that mark the boundaries of transcript isoforms. By comparing the relative abundance of long and short 3′ UTR isoforms across conditions, these tools can infer APA events such as APA switching or 3′ UTR lengthening/shortening. Several of these tools are described in detail below, with a full comparison presented in Supplementary Table S1.

DaPars is a sophisticated tool for detecting *de novo* dynamic APAs from standard RNA-seq data without the need for annotated PASs. It identifies and quantifies dynamic APA events through the comparison of RNA-seq RD between conditions. DaPars first determines a distal PAS based on coverage data and then employs a regression model to estimate the location of the proximal PAS that best fits the observed data. This approach was successfully applied to identify 1346 recurrent and tumor-specific APA events across 358 tumor-normal pairs from seven cancer types in the TCGA Pan-Cancer dataset.

APAIQ is a deep learning-based tool that enables transcriptome-wide prediction and quantification of PAS usage. It employs a hybrid deep learning model that comprises two parallel convolutional neural networks: one processes DNA sequence features, while the other processes RNA-seq coverage profiles. Both inputs undergo processing through convolutional layers and group normalization, with the rectified linear unit activation function applied to the normalized outputs. The two features are then concatenated and fed into another fully connected layer, with the final output being a PAS prediction score ranging between 0 and 1.

TAPAS is designed to handle genes with multiple APA sites, including those that occur upstream of the last exon. It builds on change-point detection algorithms from time-series analysis and incorporates additional filtering strategies to eliminate false-positive (FP) APA sites. TAPAS further supports differential APA analysis, identifying APA events that exhibit significant changes in 3′ UTR usage between conditions.

The REPAC package leverages the recount3 infrastructure to eliminate the need for raw data acquisition and preprocessing, allowing efficient analysis of APA using processed RNA-seq summaries. It quantifies differential PAS usage by computing the fold-change (cFC), which represents log-ratio-transformed changes in PAS usage across conditions. Because cFC operates in the simplex space and can be challenging to interpret directly, REPAC also reports the mean composition changes across groups, providing an interpretable estimate of average PAS usage shifts.

PolyAMiner-Bulk is an attention-based deep learning algorithm specifically designed to model APA dynamics. It captures complex PAS sequence patterns (C/PAS grammar), resolves overlapping PASs, and distinguishes nonproximal-to-distal APA changes. PolyAMiner-Bulk accounts for all APA changes, including nonproximal to nondistal changes, and can distinguish the most distal to most proximal changes from most distal to intermediate site changes irrespective of absolute change magnitude. It also offers robust visualization modules for exploring APA landscapes.

These technologies facilitate a granular examination of APA dynamics, providing insights into transcriptome diversity, cellular heterogeneity, and gene regulatory mechanisms. Their methodological diversity reflects the rapid evolution of APA analysis and enhances our ability to characterize transcriptomic complexity in diverse biological systems.

## Benchmarking analysis of current APA detection methods

### Materials and methods

We downloaded five raw RNA-seq datasets from the Gene Expression Omnibus (GEO) hosted by the National Center for Biotechnology Information (NCBI) (accession nos. GSE252630, GSE273886, GSE278174, GSE264075, and GSE251905) (see Supplementary Table S2). These datasets comprise a total of 79 ccRCC samples and 62 adjacent normal tissue samples. The reads were aligned to the human reference genome (hg38) using hisat2 (version 2.2.1) [49] with the following parameters: hisat2 -p 12 -t -x hg38_index -1$<file1 > .fastq -2$<file2 > .fastq -S < file > .sam. Aligned SAM files were then converted to BAM format and sorted using Samtools (version 1.21) [50] to facilitate downstream APA analysis.

For tool benchmarking, we selected nine APA detection tools published within the past 5 years: APAlyzer, Dapars2, APA-Scan, flexiMAP, diffUTR, TAPAS, REPAC, APAIQ, and PolyAMiner-Bulk. These tools were categorized according to their primary function: PAS detection tools, which include Dapars2, TAPAS, REPAC, APAIQ, and PolyAMiner-Bulk, and differential APA detection tools, which include APAlyzer, Dapars2, APA-Scan, flexiMAP, diffUTR, TAPAS, and REPAC. Notably, the differential APA detection module in Dapars2 builds upon the original DaPars algorithm. The detailed execution process and parameter settings of the nine tools included in the final benchmarking are outlined in the Supplementary Note S2 and Supplementary Table S7.

To evaluate the accuracy of PAS predictions, we gathered reference PAS annotations from three widely used PAS databases for human: PolyASite_2.0 [51], PolyA_DB3 [52], and GENCODE.v39 [53], all of which provide essential PAS location and gene annotation information (Supplementary Note S1). A predicted PAS was considered a true positive (TP) if it fell within a specified distance (30 bp) of a reference PAS. Precision is defined as *Precision* = $TP/\ ( {TP + FP} )$. For sensitivity calculations, overlapping predictions for the same reference PAS were counted as a single TP. Precision and sensitivity metrics were computed across various distance thresholds and gene categories.

In addition to genome-wide evaluation, we classified genes based on PAS complexity (single- versus multiple-PAS genes) to evaluate tool performance in each subgroup. The classification was based on UTR-annotated PASs compiled from the aforementioned databases.

To explore biological relevance, we further identified consensus PASs detected by at least three different tools and annotated their gene locations using PAVIS (https://manticore.niehs.nih.gov/pavis2) [54]. Functional enrichment analysis of genes containing consensus PASs was conducted using KOBAS (http://bioinfo.org/kobas/) [55]. Additionally, we analyzed six poly(A)-enriched RNA-seq data from ccRCC (accession no. GSE207574) and utilized them as a background data to evaluate the performance of differential APA detection tools. Genes with significant APA events (adjusted *P*-value < .05) detected by at least three tools were considered high-confidence candidates and included in downstream pathway enrichment analysis.

### PAS identification performance across tools

**Figure 2.**
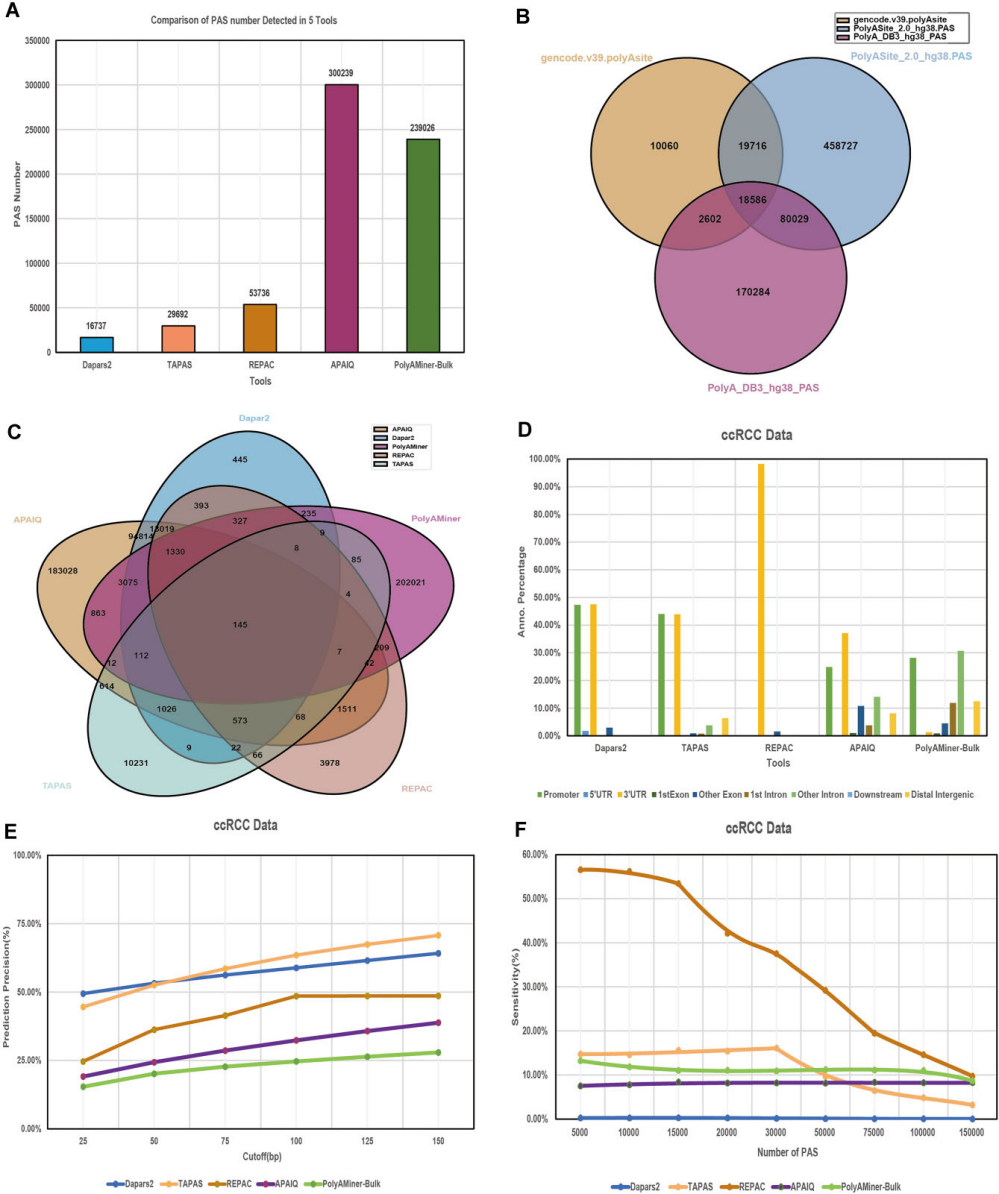
PAS detection and precision in different tools. Total number of PAS detected by different tools (**A**). The *x*-axis represents the software, and the *y*-axis indicates the number of detected PAS. Venn diagram showing the overlap of PAS (18 586) between different PAS database (**B**). Venn diagram illustrating the shared PAS (257) among the five tools (**C**). Gene locations of PAS detection by five tools (**D**). Precision of APA site prediction using different tools (**E**). Cutoffs ranging 25–150 bp in a 25-bp increment were used to determine whether a predicted poly (A) site is a TP or FP. Sensitivity of APA site prediction using different tools (**F**). A cutoff of 30 bp was used to determine whether a predicted poly (A) site is a TP or not. Top 5000–150 000 annotated poly (A) sites according to the supported number of reads were chosen as the reference for calculating sensitivity. Detailed information is provided in Supplementary Table S3 and S4.

We compared the number and characteristics of PASs identified by representative tools—DaPars2, APA-Scan, TAPAS, APAIQ, and PolyAMiner-Bulk in 79 ccRCC samples. As shown in Fig. 2A, the total number of predicted PASs varied substantially between tools: Darpars2 identified 16 737 PASs, while APAIQ predicted as many as 300 239 PASs. We found that APAIQ and PolyAMiner-Bulk, which both utilized deep learning models, generated significantly more PASs compared with alignment-based methods such as Darpars2, TAPAS, and REPAC.

To assess the consistency of PAS predictions, we analyzed the overlap among the five tools. Only 145 PASs were commonly detected by all five methods (Fig. 2C), underscoring substantial variability in predictions. Similarly, we examined overlap across three human PAS reference datasets and found only 18 586 shared PASs (Fig. 2B), likely reflecting differences in sample origins and sequencing protocols, and annotation criteria.

We also annotated the genomic distribution of PASs identified by each tool (Fig. 2D). Apart from PolyAMiner-Bulk, most tools predominantly annotated PASs to the promoter and 3′ UTR regions, suggesting a shared bias toward canonical transcript boundaries.

In terms of accuracy, the performance of TAPAS was better than that of the other methods, followed by DaPars2 and REPAC (Fig. 2E and Supplementary Table S3). Notably, when the threshold was set to 25 bp, DaPars2 exhibited the highest accuracy, while at a threshold of 50 bp, TAPAS and DaPars2 performed comparably. Sensitivity was assessed based on the number of successfully recovered annotated PASs. Among the top 30 000 reference PASs, ranked by read support, REPAC consistently obtained the highest sensitivity among all the tools, while DaPars2 had the lowest (Fig. 2F and Supplementary Table S4). These findings highlight the trade-off between precision and sensitivity among tools and the influence of prediction strategy—annotation-based, density-based, or deep learning—on performance.

### PAS detection accuracy in single- versus multi-PAS genes

**Figure 3.**
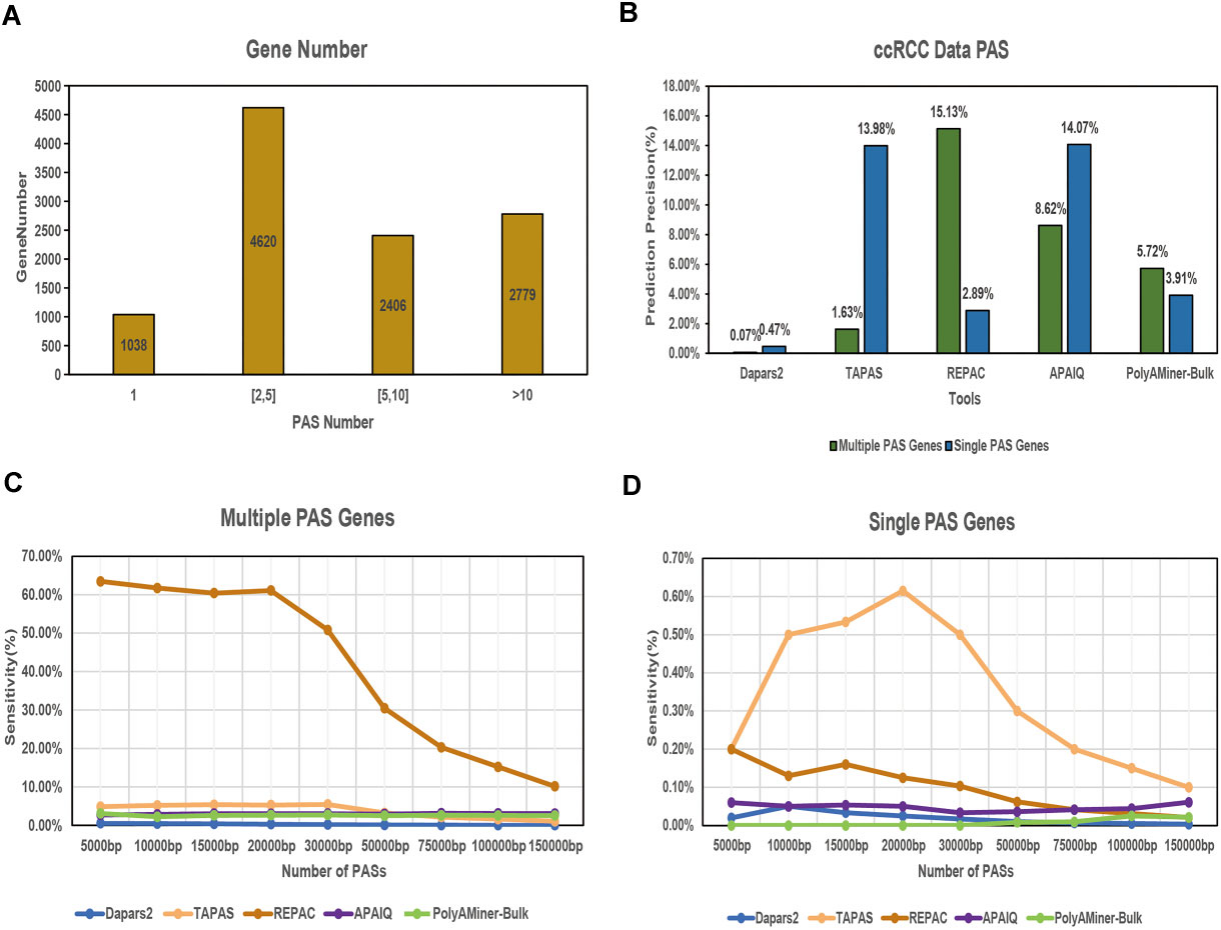
PAS precision and sensitivity in single-/multi-PAS genes. Proportion of different PAS number type genes, cutoff is 1 (single-PAS genes), between 2 and 5, between 5 and 10, and Over 10 (**A**). Precision of different tools in detecting single- and multi-PAS genes (**B**). The *x*-axis represents the software names, and the *y*-axis indicates the number of detected PAS. Bars corresponding to multi-PAS genes and single-PAS genes are distinguished by their position in the panels and legend. Sensitivity of APA site prediction using different tools in single-PAS gene (**C**) and multi-PAS gene (**D**). A cutoff of 30 bp was used to determine whether a predicted poly (A) site is a TP or not. Top 5000–150 000 annotated poly (A) sites according to the supported number of reads were chosen as the reference for calculating sensitivity. Detailed information is provided in Supplementary Table S5.

To further investigate tool performance across genes with varying PAS complexity, we classified human genes into two categories based on PAS annotations in the 3′ UTR: single-PAS genes (harboring only one PAS) and multi-PAS genes (harboring two or more PASs). Among these, single-PAS genes accounted for 1038 while the majority (4620 genes) contained between two and five PASs (Fig. 3A).

We then extracted PAS location predicted by each tool and compared them with the reference annotations for both gene types. In the analysis of genes with single and multiple PAS, REPAC achieving the highest accuracy in detecting multiple PAS genes (15.13%). Meanwhile, TAPAS and APAIQ exhibited nearly identical accuracy for detecting single-PAS genes, with TAPAS at 13.98% and APAIQ at 14.07% (Fig. 3B).

For sensitivity evaluation, we assessed how effectively each tool recovered annotated PASs within each gene category. In single-PAS gene types, TAPAS demonstrated the highest sensitivity (Fig. 3C and Supplementary Table S6), while in multi-PAS genes, REPAC again led all tools in sensitivity metrics across all subsets (Fig. 3D and Supplementary Table S5). Conversely, DaPars2 showed the lowest sensitivity across both gene types.

These results emphasize the distinct challenges associated with predicting PASs in multi-PAS genes and demonstrate that tool performance can vary significantly depending on gene architecture. While some tools excel in high-precision identification of single PASs, others offer broader sensitivity for complex gene structures with multiple PASs.

### Different APA events in ccRCC versus adjacent normal tissues

**Figure 4.**
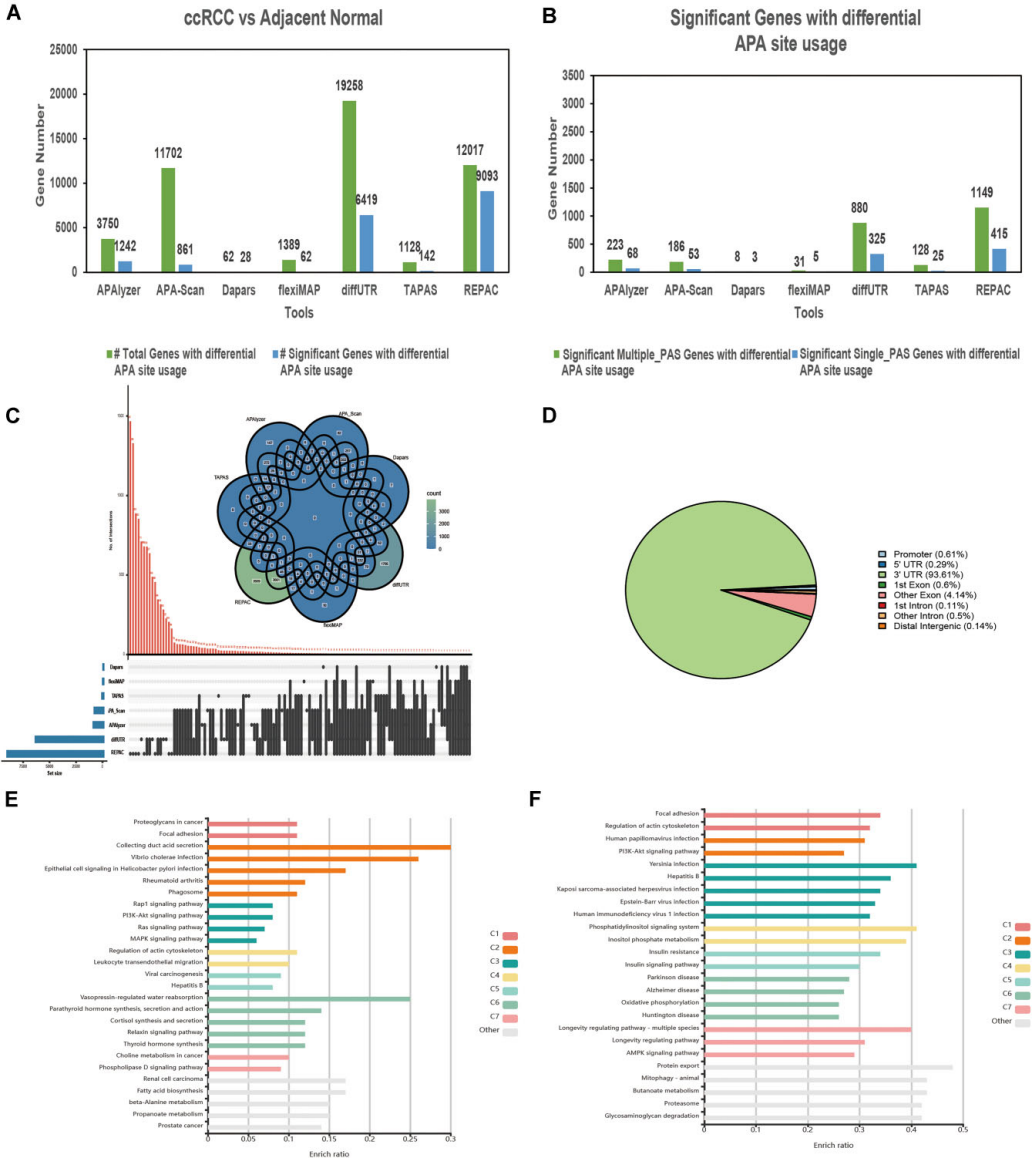
Different APA events detection in ccRCC versus adjacent normal with six tools. The total differential APA event genes and the corresponding genes with significant APA events detected by seven tools in ccRCC versus adjacent normal (**A**). The number of detected genes among single- and multi-PAS genes for the genes with significant APA events detected by seven tools (**B**). Intersection analysis was conducted on the genes with significant APA events detected by seven tools, and the results were presented using a Venn diagram (upper) and an UpSet plot (lower) (**C**). No genes appeared in the significant results of all six tools. The gene location annotation results of 145 common PAS detected by 4 PAS prediction tools (**D**). The KEGG pathway enrichment results for the genes with significant APA events that appeared in at least three APA event analysis tools (**E**) and the PAS annotated genes common to three PAS prediction tools (**F**).

To identify APA alterations associated with tumorigenesis, we compared the APA events profiles between 79 ccRCC samples and 62 adjacent normal samples using seven tools: APAlyzer, APA-Scan, diffUTR, flexiMAP, TAPAS, REPAC, and DaPars2. The number of genes detected with differential APA events varied substantially among tools (Fig. 4A). diffUTR identified the largest number of significant APA genes, followed by REPAC and APA-Scan.

To further validate tool performance, we leveraged an independent dataset comprising six poly (A)-enriched RNA-seq samples from ccRCC (GSE207574), which revealed 2379 genes with dynamic APA events detected by TAPAS, including 1750 single-PAS genes and 629 multi-PAS genes. This set was used as a reference to assess tool overlap.

Subsequently, we examined the intersection among significant APA events detected by each tool corresponded to single- versus multi-PAS genes (Fig. 4B). Notably, diffUTR identified 2835 multi-PAS genes and 220 single-PAS genes with significant differential APA, while Dapars failed to detect any single-PAS genes with statistically significant APA, highlighting its limitations in low-complexity loci.

A cross-tool intersection analysis revealed minimal overlap: No single gene was consistently identified by all tools (Fig. 4C). Most significant APA genes were uniquely reported by individual tools, reflecting the heterogeneity of algorithmic sensitivity and feature reliance. To improve result reliability, we focused on genes identified by at least three tools, yielding a high-confidence set of 866 genes for downstream analysis.

Among these, CCNL2 emerged a consistently detected APA gene identified by five tools (APAlyzer, APA-Scan, diffUTR, REPAC, and TAPAS). This suggests that APA regulation of CCNL2 may be a robust molecular event in ccRCC.

Meanwhile, we performed gene functional region annotation on the 9881 PASs that were commonly identified by at least three PAS detection tools. The majority (93.61%) of these sites were located within 3′ UTR regions (Fig. 4D), supporting the biological relevance of these predictions.

Finally, we performed KEGG pathway enrichment on two gene sets: (i) the 866 high-confidence APA-regulated genes, and (ii) the 5046 genes harboring consensus PASs. The APA-regulated genes were significantly enriched in cancer-related pathways such as Rap1 signaling pathway, PI3K–Akt signaling pathway, Ras signaling pathway, MAPK signaling pathway, and renal cell carcinoma (Fig. 4E), while the consensus PAS genes were predominantly enriched in PI3K–Akt signaling pathway and AMPK signaling pathway (Fig. 4F). These findings collectively support the regulatory significance of APA in renal tumor biology.

## Conclusions and perspective

### Biological implications of APA in cancer: insights from ccRCC

APA represents a crucial layer of post-transcriptional regulation, with 3′ UTR APA being particularly impactful in cancer biology. By modulating the length of the 3′ UTR, APA can alter the binding landscape for miRNAs and RBPs, thereby influencing mRNA stability, localization, and translational efficiency [9]. In oncogenic contexts such as ccRCC, 3′ UTR shortening may facilitate immune evasion or uncontrolled cell proliferation by removing destabilizing regulatory elements [56].

Our analysis identified CCNL2 as a recurrently APA-regulated gene in ccRCC, consistently detected by five independent tools. CCNL2 has been reported to act as a tumor suppressor, with roles in cell cycle regulation and apoptosis across various cancer types, including hepatocellular carcinoma, lung adenocarcinoma, and gastric cancer [57]. While direct mechanistic evidence linking APA to CCNL2’s function remains limited, prior studies implicating CLK1-mediated splicing and NUDT21-dependent cleavage regulation offer plausible pathways by which APA could impact its expression or isoform distribution [58, 59]. These findings underscore the potential of APA not only as a marker of disease state but also as a modifiable regulatory mechanism contributing to cancer pathogenesis.

Enrichment analysis of APA-regulated genes further supports the biological relevance of these events. Genes undergoing dynamic APA shifts were significantly enriched in signaling pathways central to tumor biology, including PI3K–Akt, Ras, MAPK, and renal cell carcinoma pathways. This suggests that APA events may play a critical role in these processes. Such convergence of APA with canonical oncogenic signaling cascades reinforces the view that APA serves as an active modulator of cellular phenotype, rather than merely a passive byproduct of transcription.

### Methodological limitations and evaluation challenges

Despite substantial advances in computational APA detection, our benchmark study revealed marked variability in the performance of available tools, reflecting differences in algorithmic assumptions, data dependencies, and target use-cases.

Our benchmarking results revealed substantial variability in the performance of APA detection tools, largely attributable to differences in algorithmic design and data dependency. Tools like REPAC, which rely heavily on pre-annotated PAS databases, exhibited the highest sensitivity but are limited in their ability to detect novel or context-specific sites. In contrast, *de novo* methods such as TAPAS and DaPars2, which infer PASs from read coverage profiles, provide broader discovery potential but at the expense of precision.

Deep learning-based tools, including APAIQ and PolyAMiner-Bulk, predicted hundreds of thousands of PASs, raising concerns about potential overcalling and FPs in the absence of orthogonal validation. Despite these differences, TAPAS and REPAC performed best overall, although both tools have limitations: TAPAS requires replicates for differential analysis, and REPAC lacks *de novo* PAS prediction capability.

The overlap of PASs predicted across tools was minimal—only 145 sites were shared among them—highlighting the influence of algorithmic strategy (annotation-based versus machine learning) and the incompleteness or redundancy of reference databases. Moreover, 3′-biased read distribution in RNA-seq and inconsistent annotation sources can further confound PAS detection, especially in methods relying on UTR coverage density.

Tool performance was also influenced by gene architecture. TAPAS showed higher precision in single-PAS genes, while REPAC performed best in multi-PAS contexts. Based on our findings, we offer the following recommendations: (i) For general PAS identification, TAPAS achieves better precision, while REPAC provides the highest sensitivity. (ii) For multi-PAS genes, REPAC is preferred; for single-PAS genes, TAPAS is more accurate. (iii) For detecting differential APA events, REPAC identifies more significant results, but we recommend integrating results from three or more tools to ensure reliability.

The accuracy of APA detection is highly dependent on sequencing depth. Tools like DaPars, which use metrics such as PDUI (percentage of distal PAS usage), rely on RD to infer APA events and are sensitive to transcript abundance. In low-depth datasets, especially in tumors where APA events may be rare, key signals may be missed. For improved resolution, high-depth RNA-seq or long-read technologies such as Iso-Seq or 3′-Seq are recommended to fully capture 3′ UTR structure and APA variability [60].

The accuracy of APA detection is closely tied to sample size. Small datasets may lack sufficient statistical power, limiting the ability to robustly identify APA events. Conversely, tools that perform well on large-scale datasets may not generalize effectively to smaller ones, where lower transcript coverage and sampling bias can reduce detection sensitivity and increase variability. This performance variability underscores the need to tailor both tool selection and analytical strategies to the specific characteristics of the dataset—particularly sample size and complexity. For example, algorithms optimized for high-throughput studies may overfit or underperform in smaller cohorts. Therefore, careful consideration of experimental design, including dataset scale, is essential to ensure optimal tool performance and reliable APA analysis.

### Conclusion and future outlook

Current APA detection tools are primarily designed to identify PASs within transcripts; however, not all APA events carry functional significance. Some may lead to unstable isoforms that are rapidly degraded, potentially serving as a regulatory mechanism for fine-tuning mRNA abundance. Several comprehensive reviews have described the biological roles, regulatory mechanisms, and detection methods of APA [26–28]. However, few have addressed the interpretability of APA results at the UTR level, nor assessed tool performance in single- versus multi-PAS gene contexts. To address these gaps, we propose several strategies for improving the reliability of PAS identification: (i) High-confidence consensus sites: Combining outputs from multiple tools and selecting overlapping PASs can enhance specificity, especially when considering the data type and sequencing platform. (ii) Multi-modal machine learning approaches: Developing new models that integrate diverse sequencing datasets—such as 3′-Seq, Iso-Seq, and bulk/single-cell RNA-seq—using deconvolution or ensemble learning frameworks may enable more robust and generalizable APA site prediction. (iii) Caution with deep learning models: Tools like APAIQ and PolyAMiner-Bulk predicted an order of magnitude more PASs than traditional methods, raising concerns about FPs. We recommend incorporating penalty mechanisms or post-hoc filtering strategies when using deep learning-based models to improve result interpretability and biological relevance.

Single-cell RNA sequencing (scRNA-seq) is a powerful technology that enables transcriptome profiling at single-cell resolution, capturing cell-to-cell transcriptional heterogeneity [61]. In recent years, several computational tools have been developed to predict PASs from scRNA-seq data, and their performance has been assessed using datasets such as those from peripheral blood mononuclear cells [26]. A major advancement in this field is the creation of single-cell APA databases—scAPAdb and scAPAatlas—which provide manually curated catalogs of poly (A) sites, APA events, and poly (A) signal motifs at the single-cell level [62, 63]. Importantly, scRNA-seq has revealed cell-type-specific APA patterns in contexts such as breast cancer, where APA regulation correlates with distinct gene expression programs across different cell types [64]. This enables the classification of cell populations based on 3′ UTR dynamics alongside transcriptomic features. As the role of APA in tumor biology becomes clearer, future studies—leveraging more accurate algorithms and higher resolution datasets—will be instrumental in elucidating APA-driven mechanisms in cancers like ccRCC, paving the way for personalized diagnostics and targeted therapies.

## Supplementary Material

lqaf056_Supplemental_File

## Data Availability

We downloaded six raw RNA-seq data from Gene Expression Omnibus (GEO) of National Center for Biotechnology Information (NCBI) (accession nos. GSE252630, GSE273886, GSE278174, GSE264075,GSE207574, and GSE251905).
