# Supplementary material for: Benchmarking of methods that identify alternative polyadenylation events in single-/multiple-polyadenylation site genes
Source: NAR Genom Bioinform. 2025 May 14;7(2):lqaf056. doi: 10.1093/nargab/lqaf056 (PMC12076406; doi:10.1093/nargab/lqaf056)
Supplement: lqaf056_Supplemental_File [file lqaf056_supplemental_file.pdf]

Supplementary Note

S1. Collection of annotated pAs

Annotated pAs of human were downloaded from GENCODE v39 (1)(n = 50,964, hg38), PolyA_DB 3 (2) (n = 301,285, Ensembl v75, hg19), and PolyASite 2.0 (3) (n = 569,005, Ensembl v96, hg38). Coordinates based on hg19 annotation were converted to hg38 using LiftOver (Genome: Ensembl v 102). Similar to (4), we then combined pAs from these databases, with the following priority: GENCODE v39 > PolyA_DB 3 > PolyASite 2.0. pAs within 24 bp of each other were grouped into pA clusters. Each cluster is considered as a non-redundant pA and the coordinate of the last pA in a cluster was considered as the coordinate of the cluster. Finally, a total of 620,996 non-redundant pAs were compiled for evaluation.

S2. Benchmarking Analysis Code Conducted in this Study

1.Dapars2

python DaPars_Extract_Anno.py -b hg38_refseq_whole_gene.bed -s hg38_Refseq_id_from_UCSC.txt -o hg38_reseq_extracted_3UTR.bed

python Dapars2_Multi_Sample.py Dapars_normol_configure.txt $chr_num

python DaPars_main.py configure_file

2.TAPAS

APA_sites_detection -cov *sample.sorted.bam.depth -ref refFlat_sf.txt -l read_length -o TAPAS_expression_with_cp_read_length.txt*

Diff_APA_site_analysis -C1 ccRCC_TAPAS_read_length.txt -C1 control_TAPAS_read_length.txt -a refFlat_sf.txt -cutoff 30 -type d

3. APAIQ

apaiq --input_file=sample.wig --out_dir=APAIQ_result --fa_file=genome.fa --name=sample --model nu398_model.ckpt --t 30

4. APAlyzer

## build Reference ranges for 3'UTR PASs in Human

download.file(url='https://ftp.ensembl.org/pub/release-113/gtf/homo_sapiens/Homo_sapiens.GRCh38.113.gtf.gz',destfile='Homo_sapiens.GRCh38.113.gtf.gz')

GTFfile="Homo_sapiens.GRCh38.113.gtf.gz"

PASREF=PAS2GEF(GTFfile, AnnoMethod="V2")

refUTRraw=PASREF$refUTRraw

```
dfIPA=PASREF$dfIPA

dfLE=PASREF$dfL

## prepare bam files

# Specify the directory containing your BAM files

bam_dir <- "bamfile_path"

# Get a list of BAM files in the specified directory

bam_files <- list.files(path = bam_dir, pattern = "\\.bam$", full.names = TRUE)

# Print the BAM files list to verify

sample_ids <- gsub(".*/(SRR[0-9]+).*", "\\1", bam_files)

#cat(paste(formatted, collapse = "\n"))

flsall <- sprintf('%s', bam_files)

names(flsall) <- gsub(".*/(SRR[0-9]+).*", "\\1", bam_files)

## PASEXP_3UTR

UTRdbraw = REF3UTR(refUTRraw)

#DFUTRraw = PASEXP_3UTR(UTRdbraw, flsall, Strandtype="forward")

batch_size <- 5

library(dplyr)

for (i in seq(1, length(flsall), by = batch_size)) {

  batch <- flsall[i:min(i + batch_size - 1, length(flsall))]

  DFUTRbatch <- PASEXP_3UTR(UTRdbraw, batch, Strandtype = "forward")

  output_filename <- paste0("DFUTR_batch_", i, "_to_", min(i + batch_size - 1, length(flsall)), ".csv")

  write.csv(DFUTRbatch, file = output_filename, row.names = TRUE)

  if (!exists("DFUTRraw")) {

    DFUTRraw <- DFUTRbatch

  } else {

    DFUTRraw <- full_join(DFUTRraw, DFUTRbatch, by = "row.names")

    rownames(DFUTRraw) <- DFUTRraw$Row.names

    DFUTRraw <- DFUTRraw[, !colnames(DFUTRraw) %in% "Row.names"]

  }

}
```

```
APAdiff(sampleTable, DFUTRraw, conKET='NT',trtKEY='KD', PAS='3UTR', CUTreads=0,
p_adjust_methods="fdr", MultiTest='unpaired t-test')
```

5. APA-scan

```
input1 = ccRCC_bamfile_path
input2 =control_bamfile_path
pas1 = NULL
pas2 = NULL
annotation = hg38_Refseq_id_from_UCSC.txt
genome = GRCh38.p14_genomic.fna
extended = no
All = no
output_dir =output_dir_path
python APA-Scan.py
```

6. flexiMAP

```
library(flexiMAP)
InputTable=read.table("input.table",sep='\t',header=T)
polyAsite=read.table("polyAsite.bed",sep='\t',header=T)
reference=read.table("reference.tsv",sep='\t',header=T)
flexiMAP(InputTable, path = "bam_file_path", covariates = "Condition", polyAsite, reference, TINfilter = NULL,
num_samples = 0.8, pairedEnd=TRUE, link = "logit", type = "ML", link.phi = "log", name = "flexiMAP_out",
normalise = FALSE, exprFilt = 5,nthread=16)
```

7. diffUTR

```
suppressPackageStartupMessages({
  library(diffUTR)
  library(SummarizedExperiment)
})
library(AnnotationHub)
ah <- AnnotationHub()
ahid <- rev(query(ah, c("EnsDb", "Homo sapiens"))$ah_id)[1]
ensdb <- ah[[ahid]]
```

```
download.file("https://polyasite.unibas.ch/download/atlas/2.0/GRCh38.96/atlas.clusters.2.0.GRCh38.96.bed.g
z",destfile = "apa.hg38.bed.gz",method = "curl")
bins <- prepareBins(ensdb, "apa.hg38.bed.gz")
rse <- countFeatures(bamfiles, bins, strandSpecific=2, nthreads=6, isPairedEnd=TRUE)
colData(rse)
condition <- c(rep("normal", number), rep("ccRCC", number))
colData(rse)$condition <- factor(condition)
colData(rse)$condition <- factor(colData(rse)$condition)
colData(rse)
rse <- diffSpliceWrapper( rse, design = ~condition )
utr <- geneLevelStats(rse, includeTypes="UTR", returnSE=FALSE)
```

8. PolyAMiner-Bulk

```
python3 PolyA-miner.py -mode bam -fasta GRCh38.p14_genomic.fna_bak -gtf GRCh38.p14_genomic.gtf
-p 20 -a 0.65 -outPrefix 3UTROnly -expNovel 1 -s 2 -o PolyAMiner-Bulk_result -c1 ccRCC_bamfile_list -c2
control_bamfile_list -ignore UTR5, CDS, Intron,UN -apriori_annotations -modelOrganism human
-visualizeTopNum 10 -visualizeCondition1Name ccRCC -visualizeCondition2Name control
```

9. REPAC

```
bams <- list.files("bam_file_path", full.names=T, pattern = "*.bam$")
data(hg38_pa)
pheno <- data.frame(groups=factor(c(rep("A", 3), rep("B",3))
rse=rse_from_bam(bams, hg38_pa, pheno, strandSpecific = 0,     allowMultiOverlap = F,
fraction = F, countMultiMappingReads=F, isPairedEnd = T, nthreads = 8,
countReadPairs = T, requireBothEndsMapped = F)
design_matrix <- model.matrix(~groups, data = colData(rse))
tb.g.corr <- fit_repac(rse, design = design_matrix)
```

Supplementary Tables

Table S1. Computational tools for detecting PAS and different APA events

Name	Description	Year	Ref	Website
Dapars	A de novo tool that directly infers the dynamic alternative polyadenylation	2014	(5)	https://github.com/ZhengXia/dapars

	(APA) usage by comparing standard RNA-seq.			
ChangePoint	A change-point model based on a likelihood ratio test for detecting 3'UTR switching.	201 4	(6)	http://utr.sourceforge.net/%20
GETUTR	Propose heuristic and regression methods to estimate and quantify the usage of 3' UTRs with widely profiled RNA sequencing (RNA-seq) data	201 5	(7)	http://big.hanyang.ac.kr/GETUTR
IsoSCM	A new method for transcript assembly, Isoform Structural Change Model (IsoSCM) that incorporates change-point analysis to improve the 3' UTR annotation process.	201 5	(8)	https://github.com/shenkers/isoscm
Roar	A strategy to identify the genes undergoing regulation of 3' UTR length using RNA sequencing data obtained from standard libraries.	201 6	(9)	https://github.com/vodkatad/roar/
QAPA	A method that infers APA from conventional RNA-seq data.	201 8	(10)	https://www.github.com/morrislab/qapa
PAQR_KAPAC	A method for quantifying poly(A) site use from RNA sequencing data and KAPAC, an approach that infers activities of oligomeric sequence motifs on poly(A) site choice.	201 8	(11)	https://github.com/zavolanlab/PAQR_KAPAC.git
APAtrap	A tool capable of refining annotated 3'UTR and identifying novel 3' UTRs and 3' UTR extensions, and aiming to identify all potential APA sites and detect genes with differential APA site usage between conditions by	201 8	(12)	https://sourceforge.net/p/apatrap/wiki/User%20Manual/

	leveraging the resolution of RNA-seq data.			
IntMap	A novel bioinformatics algorithm which integrates RNA-Seq and PolyA Site (PAS)-Seq data for a comprehensive characterization of APA events.	2018	(13)	http://compbio.cs.umn.edu/IntMAP/
MountainClimber	A de novo cumulative-sum-based approach to identify ATS and APA as change points.	2019	(14)	github.com/gxiaolab/mountainClimber
CSI-UTR	Models 3'UTR structure as tandem segments between functional alternative-polyadenylation sites (termed cleavage site intervals-CSIs).	2019	(15)	https://github.com/UofLBioinformatics/CSI-UTR
APAlyzer	A bioinformatics package for examining 3'UTR APA, intronic APA and gene expression changes using RNA-seq data and annotated polyadenylation sites in the PolyA_DB database.	2020	(16)	https://bioconductor.org/packages/release/bioc/html/APAlyzer.html
Dapars2	DaPars v2 is the next generation of DaPars that directly infers the dynamic alternative polyadenylation (APA) usage by comparing standard RNA-seq from multiple samples.	2021	(17)	https://github.com/3UTR/DaPars2
APA-Scan	A robust program that infers 3'UTR-APA events and visualizes the RNA-seq short-read coverage with gene annotations.	2022	(18)	https://github.com/compbiolabucf/APA-Scan
flexiMAP	A new beta-regression-based method implemented in R, for discovering differential alternative polyadenylation	2021	(19)	https://github.com/kszkop/flexiMAP

	events in standard RNA-seq data.			
diffUTR	The Bioconductor package which streamlines and improves upon differential exon usage (DEU) analyses, and leverages existing DEU tools and alternative poly-adenylation site databases to enable differential 3' UTR usage analysis.	2021	(20)	
TAPAS	The Translational Algorithms for Psychiatry-Advancing Science (TAPAS) software package, an open-source collection of building blocks for computational assays in psychiatry.	2021	(21)	https://github.com/arefeen/TAPAS
REPAC	A framework for the analysis of APA from RNA-sequencing data.	2023	(22)	https://github.com/eddieimada/REPAC/tree/main
APAIQ	A tool for APA identification and quantification (APAIQ) from RNA-seq data, which can accurately identify PAS and quantify PAU in a transcriptome-wide manner.	2023	(23)	https://github.com/christear/APAIQ_release
PolyAMiner-Bulk	An attention-based deep learning algorithm that accurately recapitulates C/PAS sequence grammar, resolves overlapping C/PASs, captures non-proximal-to-distal APA changes, and generates visualizations to illustrate APA dynamics.	2024	(24)	https://github.com/YalamanchiliLab/PolyAMiner-Bulk

Table S2. RNA-seq datasets used for benchmarking analysis

Run	Assay Type	Bases	BioProject	Instrument	Library Name	LibraryLayout	Organism	Sample Name	source_name	tissue
SRR28704962	RNA-Seq	6050000000	PRJNA1100931	Illumina NovaSeq 6000	GSM8209758	PAIRED	Homo sapiens	GSM8209758	kidney	kidney
SRR28704963	RNA-Seq	7090000000	PRJNA1100931	Illumina NovaSeq 6000	GSM8209757	PAIRED	Homo sapiens	GSM8209757	kidney	kidney
SRR28704964	RNA-Seq	6190000000	PRJNA1100931	Illumina NovaSeq 6000	GSM8209756	PAIRED	Homo sapiens	GSM8209756	kidney	kidney
SRR28704965	RNA-Seq	5980000000	PRJNA1100931	Illumina NovaSeq 6000	GSM8209755	PAIRED	Homo sapiens	GSM8209755	kidney	kidney
SRR28704966	RNA-Seq	6440000000	PRJNA1100931	Illumina NovaSeq 6000	GSM8209754	PAIRED	Homo sapiens	GSM8209754	kidney	kidney
SRR28704967	RNA-Seq	6540000000	PRJNA1100931	Illumina NovaSeq 6000	GSM8209753	PAIRED	Homo sapiens	GSM8209753	kidney	kidney
SRR30116783	RNA-Seq	9940000000	PRJNA1143551	Illumina NovaSeq 6000	GSM8438710	PAIRED	Homo sapiens	GSM8438710	ffpe-tumor	ffpe-tumor
SRR30116784	RNA-Seq	10300000000	PRJNA1143551	Illumina NovaSeq 6000	GSM8438709	PAIRED	Homo sapiens	GSM8438709	ffpe-tumor	ffpe-tumor
SRR30116785	RNA-Seq	11900000000	PRJNA1143551	Illumina NovaSeq 6000	GSM8438708	PAIRED	Homo sapiens	GSM8438708	ffpe-tumor	ffpe-tumor
SRR30116786	RNA-Seq	17000000000	PRJNA1143551	Illumina NovaSeq 6000	GSM8438707	PAIRED	Homo sapiens	GSM8438707	ffpe-tumor	ffpe-tumor
SRR30116787	RNA-Seq	12300000000	PRJNA1143551	Illumina NovaSeq 6000	GSM8438706	PAIRED	Homo sapiens	GSM8438706	ffpe-tumor	ffpe-tumor
SRR30116788	RNA-Seq	12500000000	PRJNA1143551	Illumina NovaSeq 6000	GSM8438705	PAIRED	Homo sapiens	GSM8438705	ffpe-tumor	ffpe-tumor
SRR30116789	RNA-Seq	11800000000	PRJNA1143551	Illumina NovaSeq 6000	GSM8438704	PAIRED	Homo sapiens	GSM8438704	ffpe-tumor	ffpe-tumor
SRR30116790	RNA-Seq	9570000000	PRJNA1143551	Illumina NovaSeq 6000	GSM8438703	PAIRED	Homo sapiens	GSM8438703	ffpe-tumor	ffpe-tumor
SRR30116791	RNA-Seq	11600000000	PRJNA1143551	Illumina NovaSeq 6000	GSM8438702	PAIRED	Homo sapiens	GSM8438702	ffpe-tumor	ffpe-tumor
SRR30116792	RNA-Seq	11400000000	PRJNA1143551	Illumina NovaSeq 6000	GSM8438701	PAIRED	Homo sapiens	GSM8438701	ffpe-tumor	ffpe-tumor
SRR30116793	RNA-Seq	8800000000	PRJNA1143551	Illumina NovaSeq 6000	GSM8438700	PAIRED	Homo sapiens	GSM8438700	ffpe-tumor	ffpe-tumor
SRR30116794	RNA-Seq	9910000000	PRJNA1143551	Illumina NovaSeq 6000	GSM8438699	PAIRED	Homo sapiens	GSM8438699	ffpe-tumor	ffpe-tumor
SRR30116795	RNA-Seq	10000000000	PRJNA1143551	Illumina NovaSeq 6000	GSM8438698	PAIRED	Homo sapiens	GSM8438698	ffpe-tumor	ffpe-tumor
SRR30116796	RNA-Seq	11800000000	PRJNA1143551	Illumina NovaSeq 6000	GSM8438697	PAIRED	Homo sapiens	GSM8438697	ffpe-tumor	ffpe-tumor
SRR30116797	RNA-Seq	12800000000	PRJNA1143551	Illumina NovaSeq 6000	GSM8438696	PAIRED	Homo sapiens	GSM8438696	ffpe-tumor	ffpe-tumor

SRR30116798	RNA-Seq	11000000000	PRJNA1143551	Illumina NovaSeq 6000	GSM8438695	PAIRED	Homo sapiens	GSM8438695	ffpe-tumor	ffpe-tumor
SRR30812727	RNA-Seq	6230000000	PRJNA1165804	Illumina HiSeq 2500	GSM8541106	PAIRED	Homo sapiens	GSM8541106	Clear Cell RCC	Clear Cell RCC
SRR30812728	RNA-Seq	6970000000	PRJNA1165804	Illumina HiSeq 2500	GSM8541105	PAIRED	Homo sapiens	GSM8541105	Clear Cell RCC	Clear Cell RCC
SRR30812729	RNA-Seq	1520000000	PRJNA1165804	Illumina HiSeq 2500	GSM8541104	PAIRED	Homo sapiens	GSM8541104	Clear Cell RCC	Clear Cell RCC
SRR30812730	RNA-Seq	1370000000	PRJNA1165804	Illumina HiSeq 2500	GSM8541103	PAIRED	Homo sapiens	GSM8541103	Clear Cell RCC	Clear Cell RCC
SRR30812731	RNA-Seq	2010000000	PRJNA1165804	Illumina HiSeq 2500	GSM8541102	PAIRED	Homo sapiens	GSM8541102	Clear Cell RCC	Clear Cell RCC
SRR30812732	RNA-Seq	7720000000	PRJNA1165804	Illumina HiSeq 2500	GSM8541101	PAIRED	Homo sapiens	GSM8541101	Clear Cell RCC	Clear Cell RCC
SRR30812733	RNA-Seq	3370000000	PRJNA1165804	Illumina HiSeq 2500	GSM8541100	PAIRED	Homo sapiens	GSM8541100	Clear Cell RCC	Clear Cell RCC
SRR30812734	RNA-Seq	4060000000	PRJNA1165804	Illumina HiSeq 2500	GSM8541099	PAIRED	Homo sapiens	GSM8541099	Clear Cell RCC	Clear Cell RCC
SRR30812735	RNA-Seq	3550000000	PRJNA1165804	Illumina HiSeq 2500	GSM8541098	PAIRED	Homo sapiens	GSM8541098	Clear Cell RCC	Clear Cell RCC
SRR30812736	RNA-Seq	2950000000	PRJNA1165804	Illumina HiSeq 2500	GSM8541097	PAIRED	Homo sapiens	GSM8541097	Clear Cell RCC	Clear Cell RCC
SRR30812737	RNA-Seq	6560000000	PRJNA1165804	Illumina HiSeq 2500	GSM8541096	PAIRED	Homo sapiens	GSM8541096	Clear Cell RCC	Clear Cell RCC
SRR30812738	RNA-Seq	6210000000	PRJNA1165804	Illumina HiSeq 2500	GSM8541095	PAIRED	Homo sapiens	GSM8541095	Clear Cell RCC	Clear Cell RCC
SRR30812739	RNA-Seq	8170000000	PRJNA1165804	Illumina HiSeq 2500	GSM8541094	PAIRED	Homo sapiens	GSM8541094	Clear Cell RCC	Clear Cell RCC
SRR30812740	RNA-Seq	4190000000	PRJNA1165804	Illumina HiSeq 2500	GSM8541093	PAIRED	Homo sapiens	GSM8541093	Clear Cell RCC	Clear Cell RCC
SRR30812741	RNA-Seq	5740000000	PRJNA1165804	Illumina HiSeq 2500	GSM8541092	PAIRED	Homo sapiens	GSM8541092	Clear Cell RCC	Clear Cell RCC
SRR30812742	RNA-Seq	7060000000	PRJNA1165804	Illumina HiSeq 2500	GSM8541091	PAIRED	Homo sapiens	GSM8541091	Clear Cell RCC	Clear Cell RCC
SRR30812743	RNA-Seq	8630000000	PRJNA1165804	Illumina HiSeq 2500	GSM8541090	PAIRED	Homo sapiens	GSM8541090	Clear Cell RCC	Clear Cell RCC
SRR30812744	RNA-Seq	8850000000	PRJNA1165804	Illumina HiSeq 2500	GSM8541089	PAIRED	Homo sapiens	GSM8541089	Clear Cell RCC	Clear Cell RCC
SRR30812745	RNA-Seq	8420000000	PRJNA1165804	Illumina HiSeq 2500	GSM8541088	PAIRED	Homo sapiens	GSM8541088	Clear Cell RCC	Clear Cell RCC
SRR27437837	RNA-Seq	6540000000	PRJNA1061486	Illumina HiSeq 2500	GSM8003781	PAIRED	Homo sapiens	GSM8003781	kidney	normal
SRR27437838	RNA-Seq	6570000000	PRJNA1061486	Illumina HiSeq 2500	GSM8003780	PAIRED	Homo sapiens	GSM8003780	kidney	normal
SRR27437839	RNA-Seq	6800000000	PRJNA1061486	Illumina HiSeq 2500	GSM8003779	PAIRED	Homo sapiens	GSM8003779	kidney	normal
SRR27437840	RNA-Seq	8480000000	PRJNA1061486	Illumina HiSeq 2500	GSM8003778	PAIRED	Homo sapiens	GSM8003778	kidney	normal

SRR27437841	RNA-Seq	6570000000	PRJNA1061486	Illumina HiSeq 2500	GSM8003777	PAIRED	Homo sapiens	GSM8003777	kidney	normal
SRR27437842	RNA-Seq	7130000000	PRJNA1061486	Illumina HiSeq 2500	GSM8003776	PAIRED	Homo sapiens	GSM8003776	kidney	normal
SRR27437843	RNA-Seq	6320000000	PRJNA1061486	Illumina HiSeq 2500	GSM8003775	PAIRED	Homo sapiens	GSM8003775	kidney	normal
SRR27437844	RNA-Seq	7140000000	PRJNA1061486	Illumina HiSeq 2500	GSM8003774	PAIRED	Homo sapiens	GSM8003774	kidney	normal
SRR27437845	RNA-Seq	9460000000	PRJNA1061486	Illumina HiSeq 2500	GSM8003773	PAIRED	Homo sapiens	GSM8003773	kidney	normal
SRR27437846	RNA-Seq	8650000000	PRJNA1061486	Illumina HiSeq 2500	GSM8003772	PAIRED	Homo sapiens	GSM8003772	kidney	tumor stage 4
SRR27437847	RNA-Seq	7400000000	PRJNA1061486	Illumina HiSeq 2500	GSM8003771	PAIRED	Homo sapiens	GSM8003771	kidney	tumor stage 2
SRR27437848	RNA-Seq	8580000000	PRJNA1061486	Illumina HiSeq 2500	GSM8003770	PAIRED	Homo sapiens	GSM8003770	kidney	tumor stage 4
SRR27437849	RNA-Seq	7340000000	PRJNA1061486	Illumina HiSeq 2500	GSM8003769	PAIRED	Homo sapiens	GSM8003769	kidney	tumor stage 3
SRR27437850	RNA-Seq	7570000000	PRJNA1061486	Illumina HiSeq 2500	GSM8003768	PAIRED	Homo sapiens	GSM8003768	kidney	tumor stage 1
SRR27437851	RNA-Seq	7930000000	PRJNA1061486	Illumina HiSeq 2500	GSM8003767	PAIRED	Homo sapiens	GSM8003767	kidney	tumor stage 1
SRR27437852	RNA-Seq	7120000000	PRJNA1061486	Illumina HiSeq 2500	GSM8003766	PAIRED	Homo sapiens	GSM8003766	kidney	tumor stage 3
SRR27437853	RNA-Seq	8630000000	PRJNA1061486	Illumina HiSeq 2500	GSM8003765	PAIRED	Homo sapiens	GSM8003765	kidney	tumor stage 1
SRR27437854	RNA-Seq	6070000000	PRJNA1061486	Illumina HiSeq 2500	GSM8003764	PAIRED	Homo sapiens	GSM8003764	kidney	tumor stage 2
SRR27329330	RNA-Seq	3780000000	PRJNA1055903	NextSeq 500	GSM7989821	SINGLE	Homo sapiens	GSM7989821	ccRCC Primary Tumour	ccRCC Primary Tumour
SRR27329333	RNA-Seq	2100000000	PRJNA1055903	NextSeq 500	GSM7989818	SINGLE	Homo sapiens	GSM7989818	ccRCC Primary Tumour	ccRCC Primary Tumour
SRR27329334	RNA-Seq	2430000000	PRJNA1055903	NextSeq 500	GSM7989817	SINGLE	Homo sapiens	GSM7989817	Adjacent Kidney	Adjacent Kidney
SRR27329336	RNA-Seq	3000000000	PRJNA1055903	NextSeq 500	GSM7989815	SINGLE	Homo sapiens	GSM7989815	ccRCC Primary Tumour	ccRCC Primary Tumour
SRR27329337	RNA-Seq	3180000000	PRJNA1055903	NextSeq 500	GSM7989814	SINGLE	Homo sapiens	GSM7989814	Adjacent Kidney	Adjacent Kidney
SRR27329339	RNA-Seq	2570000000	PRJNA1055903	NextSeq 500	GSM7989812	SINGLE	Homo sapiens	GSM7989812	ccRCC Primary Tumour	ccRCC Primary Tumour
SRR27329340	RNA-Seq	2030000000	PRJNA1055903	NextSeq 500	GSM7989811	SINGLE	Homo sapiens	GSM7989811	Adjacent Kidney	Adjacent Kidney
SRR27329345	RNA-Seq	3100000000	PRJNA1055903	NextSeq 500	GSM7989790	SINGLE	Homo sapiens	GSM7989790	ccRCC Primary Tumour	ccRCC Primary Tumour
SRR27329346	RNA-Seq	2280000000	PRJNA1055903	NextSeq 500	GSM7989789	SINGLE	Homo sapiens	GSM7989789	ccRCC Primary Tumour	ccRCC Primary Tumour
SRR27329347	RNA-Seq	2410000000	PRJNA1055903	NextSeq 500	GSM7989788	SINGLE	Homo sapiens	GSM7989788	ccRCC Primary Tumour	ccRCC Primary Tumour

SRR27329348	RNA-Seq	2380000000	PRJNA1055903	NextSeq 500	GSM7989787	SINGLE	Homo sapiens	GSM7989787	ccRCC Primary Tumour	ccRCC Primary Tumour
SRR27329349	RNA-Seq	2350000000	PRJNA1055903	NextSeq 500	GSM7989786	SINGLE	Homo sapiens	GSM7989786	ccRCC Primary Tumour	ccRCC Primary Tumour
SRR27329350	RNA-Seq	2190000000	PRJNA1055903	NextSeq 500	GSM7989785	SINGLE	Homo sapiens	GSM7989785	ccRCC Primary Tumour	ccRCC Primary Tumour
SRR27329351	RNA-Seq	2580000000	PRJNA1055903	NextSeq 500	GSM7989784	SINGLE	Homo sapiens	GSM7989784	ccRCC Primary Tumour	ccRCC Primary Tumour
SRR27329352	RNA-Seq	1110000000	PRJNA1055903	NextSeq 500	GSM7989783	SINGLE	Homo sapiens	GSM7989783	ccRCC Primary Tumour	ccRCC Primary Tumour
SRR27329353	RNA-Seq	4110000000	PRJNA1055903	NextSeq 500	GSM7989782	SINGLE	Homo sapiens	GSM7989782	ccRCC Primary Tumour	ccRCC Primary Tumour
SRR27329354	RNA-Seq	2680000000	PRJNA1055903	NextSeq 500	GSM7989781	SINGLE	Homo sapiens	GSM7989781	ccRCC Primary Tumour	ccRCC Primary Tumour
SRR27329355	RNA-Seq	3300000000	PRJNA1055903	NextSeq 500	GSM7989780	SINGLE	Homo sapiens	GSM7989780	ccRCC Primary Tumour	ccRCC Primary Tumour
SRR27329356	RNA-Seq	3480000000	PRJNA1055903	NextSeq 500	GSM7989779	SINGLE	Homo sapiens	GSM7989779	ccRCC Primary Tumour	ccRCC Primary Tumour
SRR27329357	RNA-Seq	3510000000	PRJNA1055903	NextSeq 500	GSM7989778	SINGLE	Homo sapiens	GSM7989778	ccRCC Primary Tumour	ccRCC Primary Tumour
SRR27329358	RNA-Seq	2470000000	PRJNA1055903	NextSeq 500	GSM7989777	SINGLE	Homo sapiens	GSM7989777	ccRCC Primary Tumour	ccRCC Primary Tumour
SRR27329359	RNA-Seq	2330000000	PRJNA1055903	NextSeq 500	GSM7989776	SINGLE	Homo sapiens	GSM7989776	ccRCC Primary Tumour	ccRCC Primary Tumour
SRR27329360	RNA-Seq	2690000000	PRJNA1055903	NextSeq 500	GSM7989775	SINGLE	Homo sapiens	GSM7989775	ccRCC Primary Tumour	ccRCC Primary Tumour
SRR27329362	RNA-Seq	2710000000	PRJNA1055903	NextSeq 500	GSM7989830	SINGLE	Homo sapiens	GSM7989830	ccRCC Primary Tumour	ccRCC Primary Tumour
SRR27329363	RNA-Seq	3030000000	PRJNA1055903	NextSeq 500	GSM7989829	SINGLE	Homo sapiens	GSM7989829	Adjacent Kidney	Adjacent Kidney
SRR27329365	RNA-Seq	3250000000	PRJNA1055903	NextSeq 500	GSM7989827	SINGLE	Homo sapiens	GSM7989827	ccRCC Primary Tumour	ccRCC Primary Tumour
SRR27329366	RNA-Seq	3300000000	PRJNA1055903	NextSeq 500	GSM7989826	SINGLE	Homo sapiens	GSM7989826	Adjacent Kidney	Adjacent Kidney
SRR27329368	RNA-Seq	3110000000	PRJNA1055903	NextSeq 500	GSM7989824	SINGLE	Homo sapiens	GSM7989824	ccRCC Primary Tumour	ccRCC Primary Tumour
SRR27329369	RNA-Seq	2570000000	PRJNA1055903	NextSeq 500	GSM7989823	SINGLE	Homo sapiens	GSM7989823	Adjacent Kidney	Adjacent Kidney
SRR27329384	RNA-Seq	2880000000	PRJNA1055903	NextSeq 500	GSM7989792	SINGLE	Homo sapiens	GSM7989792	ccRCC Primary Tumour	ccRCC Primary Tumour
SRR27329385	RNA-Seq	3400000000	PRJNA1055903	NextSeq 500	GSM7989791	SINGLE	Homo sapiens	GSM7989791	ccRCC Primary Tumour	ccRCC Primary Tumour
SRR27329386	RNA-Seq	2670000000	PRJNA1055903	NextSeq 500	GSM7989774	SINGLE	Homo sapiens	GSM7989774	ccRCC Primary Tumour	ccRCC Primary Tumour
SRR27329387	RNA-Seq	2760000000	PRJNA1055903	NextSeq 500	GSM7989773	SINGLE	Homo sapiens	GSM7989773	ccRCC Primary Tumour	ccRCC Primary Tumour
SRR27329388	RNA-Seq	2270000000	PRJNA1055903	NextSeq 500	GSM7989772	SINGLE	Homo sapiens	GSM7989772	ccRCC Primary Tumour	ccRCC Primary Tumour

SRR27329389	RNA-Seq	1790000000	PRJNA1055903	NextSeq 500	GSM7989771	SINGLE	Homo sapiens	GSM7989771	ccRCC Primary Tumour	ccRCC Primary Tumour
SRR27329390	RNA-Seq	2430000000	PRJNA1055903	NextSeq 500	GSM7989770	SINGLE	Homo sapiens	GSM7989770	ccRCC Primary Tumour	ccRCC Primary Tumour
SRR27329391	RNA-Seq	3030000000	PRJNA1055903	NextSeq 500	GSM7989769	SINGLE	Homo sapiens	GSM7989769	ccRCC Primary Tumour	ccRCC Primary Tumour
SRR27329392	RNA-Seq	3080000000	PRJNA1055903	NextSeq 500	GSM7989768	SINGLE	Homo sapiens	GSM7989768	ccRCC Primary Tumour	ccRCC Primary Tumour
SRR27329402	RNA-Seq	2000000000	PRJNA1055903	NextSeq 500	GSM7989742	SINGLE	Homo sapiens	GSM7989742	Adjacent Kidney	Adjacent Kidney
SRR27329403	RNA-Seq	2480000000	PRJNA1055903	NextSeq 500	GSM7989741	SINGLE	Homo sapiens	GSM7989741	Adjacent Kidney	Adjacent Kidney
SRR27329404	RNA-Seq	1710000000	PRJNA1055903	NextSeq 500	GSM7989740	SINGLE	Homo sapiens	GSM7989740	Adjacent Kidney	Adjacent Kidney
SRR27329405	RNA-Seq	5280000000	PRJNA1055903	NextSeq 500	GSM7989739	SINGLE	Homo sapiens	GSM7989739	Adjacent Kidney	Adjacent Kidney
SRR27329406	RNA-Seq	1810000000	PRJNA1055903	NextSeq 500	GSM7989738	SINGLE	Homo sapiens	GSM7989738	Adjacent Kidney	Adjacent Kidney
SRR27329407	RNA-Seq	3250000000	PRJNA1055903	NextSeq 500	GSM7989737	SINGLE	Homo sapiens	GSM7989737	Adjacent Kidney	Adjacent Kidney
SRR27329408	RNA-Seq	2760000000	PRJNA1055903	NextSeq 500	GSM7989736	SINGLE	Homo sapiens	GSM7989736	Adjacent Kidney	Adjacent Kidney
SRR27329409	RNA-Seq	3250000000	PRJNA1055903	NextSeq 500	GSM7989735	SINGLE	Homo sapiens	GSM7989735	Adjacent Kidney	Adjacent Kidney
SRR27329410	RNA-Seq	2170000000	PRJNA1055903	NextSeq 500	GSM7989734	SINGLE	Homo sapiens	GSM7989734	Adjacent Kidney	Adjacent Kidney
SRR27329411	RNA-Seq	795000000	PRJNA1055903	NextSeq 500	GSM7989733	SINGLE	Homo sapiens	GSM7989733	Adjacent Kidney	Adjacent Kidney
SRR27329412	RNA-Seq	3110000000	PRJNA1055903	NextSeq 500	GSM7989732	SINGLE	Homo sapiens	GSM7989732	Adjacent Kidney	Adjacent Kidney
SRR27329413	RNA-Seq	2860000000	PRJNA1055903	NextSeq 500	GSM7989731	SINGLE	Homo sapiens	GSM7989731	Adjacent Kidney	Adjacent Kidney
SRR27329414	RNA-Seq	2960000000	PRJNA1055903	NextSeq 500	GSM7989730	SINGLE	Homo sapiens	GSM7989730	Adjacent Kidney	Adjacent Kidney
SRR27329415	RNA-Seq	2120000000	PRJNA1055903	NextSeq 500	GSM7989729	SINGLE	Homo sapiens	GSM7989729	Adjacent Kidney	Adjacent Kidney
SRR27329416	RNA-Seq	2760000000	PRJNA1055903	NextSeq 500	GSM7989728	SINGLE	Homo sapiens	GSM7989728	Adjacent Kidney	Adjacent Kidney
SRR27329417	RNA-Seq	2500000000	PRJNA1055903	NextSeq 500	GSM7989727	SINGLE	Homo sapiens	GSM7989727	Adjacent Kidney	Adjacent Kidney
SRR27329418	RNA-Seq	2660000000	PRJNA1055903	NextSeq 500	GSM7989726	SINGLE	Homo sapiens	GSM7989726	Adjacent Kidney	Adjacent Kidney
SRR27329419	RNA-Seq	1420000000	PRJNA1055903	NextSeq 500	GSM7989725	SINGLE	Homo sapiens	GSM7989725	Adjacent Kidney	Adjacent Kidney
SRR27329420	RNA-Seq	4670000000	PRJNA1055903	NextSeq 500	GSM7989724	SINGLE	Homo sapiens	GSM7989724	Adjacent Kidney	Adjacent Kidney
SRR27329421	RNA-Seq	2170000000	PRJNA1055903	NextSeq 500	GSM7989723	SINGLE	Homo sapiens	GSM7989723	Adjacent Kidney	Adjacent Kidney

SRR27329422	RNA-Seq	3230000000	PRJNA1055903	NextSeq 500	GSM7989722	SINGLE	Homo sapiens	GSM7989722	Adjacent Kidney	Adjacent Kidney
SRR27329423	RNA-Seq	1910000000	PRJNA1055903	NextSeq 500	GSM7989721	SINGLE	Homo sapiens	GSM7989721	Adjacent Kidney	Adjacent Kidney
SRR27329424	RNA-Seq	3040000000	PRJNA1055903	NextSeq 500	GSM7989720	SINGLE	Homo sapiens	GSM7989720	Adjacent Kidney	Adjacent Kidney
SRR27329425	RNA-Seq	2960000000	PRJNA1055903	NextSeq 500	GSM7989719	SINGLE	Homo sapiens	GSM7989719	Adjacent Kidney	Adjacent Kidney
SRR27329426	RNA-Seq	2130000000	PRJNA1055903	NextSeq 500	GSM7989718	SINGLE	Homo sapiens	GSM7989718	Adjacent Kidney	Adjacent Kidney
SRR27329427	RNA-Seq	1700000000	PRJNA1055903	NextSeq 500	GSM7989717	SINGLE	Homo sapiens	GSM7989717	Adjacent Kidney	Adjacent Kidney
SRR27329428	RNA-Seq	1800000000	PRJNA1055903	NextSeq 500	GSM7989716	SINGLE	Homo sapiens	GSM7989716	Adjacent Kidney	Adjacent Kidney
SRR27329429	RNA-Seq	2890000000	PRJNA1055903	NextSeq 500	GSM7989715	SINGLE	Homo sapiens	GSM7989715	Adjacent Kidney	Adjacent Kidney
SRR27329430	RNA-Seq	2680000000	PRJNA1055903	NextSeq 500	GSM7989714	SINGLE	Homo sapiens	GSM7989714	Adjacent Kidney	Adjacent Kidney
SRR27329431	RNA-Seq	1980000000	PRJNA1055903	NextSeq 500	GSM7989713	SINGLE	Homo sapiens	GSM7989713	Adjacent Kidney	Adjacent Kidney
SRR27329432	RNA-Seq	2470000000	PRJNA1055903	NextSeq 500	GSM7989712	SINGLE	Homo sapiens	GSM7989712	Adjacent Kidney	Adjacent Kidney
SRR27329433	RNA-Seq	4300000000	PRJNA1055903	NextSeq 500	GSM7989711	SINGLE	Homo sapiens	GSM7989711	Adjacent Kidney	Adjacent Kidney
SRR27329440	RNA-Seq	2920000000	PRJNA1055903	NextSeq 500	GSM7989752	SINGLE	Homo sapiens	GSM7989752	Adjacent Kidney	Adjacent Kidney
SRR27329441	RNA-Seq	1960000000	PRJNA1055903	NextSeq 500	GSM7989751	SINGLE	Homo sapiens	GSM7989751	Adjacent Kidney	Adjacent Kidney
SRR27329442	RNA-Seq	3250000000	PRJNA1055903	NextSeq 500	GSM7989750	SINGLE	Homo sapiens	GSM7989750	Adjacent Kidney	Adjacent Kidney
SRR27329443	RNA-Seq	2390000000	PRJNA1055903	NextSeq 500	GSM7989749	SINGLE	Homo sapiens	GSM7989749	Adjacent Kidney	Adjacent Kidney
SRR27329444	RNA-Seq	2350000000	PRJNA1055903	NextSeq 500	GSM7989748	SINGLE	Homo sapiens	GSM7989748	Adjacent Kidney	Adjacent Kidney
SRR27329445	RNA-Seq	2330000000	PRJNA1055903	NextSeq 500	GSM7989747	SINGLE	Homo sapiens	GSM7989747	Adjacent Kidney	Adjacent Kidney
SRR27329446	RNA-Seq	1990000000	PRJNA1055903	NextSeq 500	GSM7989746	SINGLE	Homo sapiens	GSM7989746	Adjacent Kidney	Adjacent Kidney
SRR27329447	RNA-Seq	2540000000	PRJNA1055903	NextSeq 500	GSM7989745	SINGLE	Homo sapiens	GSM7989745	Adjacent Kidney	Adjacent Kidney
SRR27329448	RNA-Seq	1610000000	PRJNA1055903	NextSeq 500	GSM7989744	SINGLE	Homo sapiens	GSM7989744	Adjacent Kidney	Adjacent Kidney
SRR27329449	RNA-Seq	2830000000	PRJNA1055903	NextSeq 500	GSM7989743	SINGLE	Homo sapiens	GSM7989743	Adjacent Kidney	Adjacent Kidney
SRR27329450	RNA-Seq	2830000000	PRJNA1055903	NextSeq 500	GSM7989710	SINGLE	Homo sapiens	GSM7989710	Adjacent Kidney	Adjacent Kidney
SRR27329451	RNA-Seq	2390000000	PRJNA1055903	NextSeq 500	GSM7989709	SINGLE	Homo sapiens	GSM7989709	Adjacent Kidney	Adjacent Kidney

Table S3 Number of predicted APA sites and true positives by applying different tools on RNA-seq datasets.

		Number of true positives at different cutoffs					
Tool	PAS number	25bp	50bp	75bp	100bp	125bp	150bp
Dapars2	16737	8275	8907	9413	9853	10299	10739
TAPAS	29692	13234	15609	17386	18856	20022	21009
REPAC	53736	13239	19479	22257	26085	26113	26128
APAIQ	300239	57405	73139	85875	97168	107319	116503
PolyAMiner-Bulk	239026	36792	48175	54300	58923	62981	66777

Table S4 Number of truly predicted APA sites by applying different tools on RNA-seq datasets.

		True positives in different reference datasets								
Tool	PAS number	5000	10000	15000	20000	30000	50000	75000	100000	150000
Dapars2	16737	12	26	43	47	47	47	47	47	47
TAPAS	29692	737	1460	2340	3078	4824	4824	4824	4824	4824
REPAC	53736	2827	5615	8009	8429	11252	14597	14597	14597	14597
APAIQ	300239	376	777	1260	1631	2456	4064	6252	8233	12373
PolyAMiner-Bulk	239026	662	1183	1652	2211	3277	5608	8342	11021	13026

Table S5 Number of truly predicted APA sites in multi-PAS genes by applying different tools on RNA-seq datasets.

Tool	5000	10000	15000	20000	30000	50000	75000	100000	150000
Dapars2	28	48	64	69	69	69	69	69	69
TAPAS	244	524	810	1063	1640	1640	1640	1640	1640
REPAC	3173	6172	9059	12215	15244	15244	15244	15244	15244
APAIQ	137	289	462	611	922	1476	2382	3126	4653
PolyAMiner-Bulk	156	228	395	537	824	1276	1991	2599	3862

Table S6 Number of truly predicted APA sites in single-PAS genes by applying different tools on RNA-seq datasets.

Tool	5000	10000	15000	20000	30000	50000	75000	100000	150000
Dapars2	1	5	5	5	5	5	5	5	5
TAPAS	10	50	80	123	150	150	150	150	150
REPAC	10	13	24	25	31	31	31	31	31

APAIQ	3	5	8	10	10	18	31	44	91
PolyAMiner-Bulk	0	0	0	0	0	4	7	25	32

Table S7 Parameter combinations of different tools in PAS prediction and APA events detection.

Tool	Details on the Parameter Settings
APAlyzer	-conKET 'NT' -trtKEY 'KD' -PAS '3UTR' -CUTreads 0 -p_adjust_methods "fdr" -MultiTest 'unpaired t-test'
Dapars2	-PDUI_cutoff 0.5 -Coverage_cutoff 10 -FDR_cutoff 0.05
APA-Scan	-Extended_3UTR no -All no -absolute_ratio_difference 0.1 -p_value 0.5
flexiMAP	-TINfilter NULL -num_samples 0.8 -pairedEnd TRUE -link "logit" -type "ML" -link.phi "log" -normalise FALSE -exprFilt 5
diffUTR	-strandSpecific 2 -isPairedEnd TRUE -includeTypes UTR
TAPAS	-l 50 -cutoff 30 -type d
REPAC	-strandSpecific 0 -allowMultiOverlap F -fraction F – countMultiMappingReads F -isPairedEnd T -countReadPairs T -requireBothEndsMapped F
APAIQ	-model nu398_model.ckpt -t 30
PolyAMiner-Bulk	-mode bam -p 20 -a 0.65 -outPrefix 3UTROnly -expNovel 1 -s 2 -ignore UTR5, CDS, Intron,UN -apriori_annotations -modelOrganism human

References

1. Frankish, A., Diekhans, M., Ferreira, A.M., Johnson, R., Jungreis, I., Loveland, J., Mudge, J.M., Sisu, C., Wright, J., Armstrong, J. *et al.* (2019) GENCODE reference annotation for the human and mouse genomes. *Nucleic Acids Res*, **47**, D766-d773.
2. Wang, R., Nambiar, R., Zheng, D. and Tian, B. (2018) PolyA_DB 3 catalogs cleavage and polyadenylation sites identified by deep sequencing in multiple genomes. *Nucleic Acids Res*, **46**, D315-d319.
3. Gruber, A.J., Schmidt, R., Gruber, A.R., Martin, G., Ghosh, S., Belmadani, M., Keller, W. and Zavolan, M. (2016) A comprehensive analysis of 3' end sequencing data sets reveals novel polyadenylation signals and the repressive role of heterogeneous ribonucleoprotein C on cleavage and polyadenylation. *Genome research*, **26**, 1145-1159.
4. Agarwal, V., Lopez-Darwin, S., Kelley, D.R. and Shendure, J. (2021) The landscape of alternative polyadenylation in single cells of the developing mouse embryo. *Nature communications*, **12**, 5101.
5. Xia, Z., Donehower, L.A., Cooper, T.A., Neilson, J.R., Wheeler, D.A., Wagner, E.J. and Li, W. (2014) Dynamic analyses of alternative polyadenylation from RNA-seq reveal a 3′-UTR landscape across seven tumour types. *Nature Communications*, **5**, 5274.
6. Wang, W., Wei, Z. and Li, H. (2014) A change-point model for identifying 3'UTR switching by next-generation RNA sequencing. *Bioinformatics*, **30**, 2162-2170.
7. Kim, M., You, B.H. and Nam, J.W. (2015) Global estimation of the 3' untranslated region landscape using RNA sequencing. *Methods*, **83**, 111-117.
8. Shenker, S., Miura, P., Sanfilippo, P. and Lai, E.C. (2015) IsoSCM: improved and alternative 3' UTR annotation using multiple change-point inference. *Rna*, **21**, 14-27.
9. Grassi, E., Mariella, E., Lembo, A., Molineris, I. and Provero, P. (2016) Roar: detecting alternative polyadenylation with standard mRNA sequencing libraries. *BMC bioinformatics*, **17**, 423.
10. Ha, K.C.H., Blencowe, B.J. and Morris, Q. (2018) QAPA: a new method for the systematic analysis of alternative polyadenylation from RNA-seq data. *Genome Biol*, **19**, 45.
11. Gruber, A.J., Schmidt, R., Ghosh, S., Martin, G., Gruber, A.R., van Nimwegen, E. and Zavolan, M. (2018) Discovery of physiological and cancer-related regulators of 3' UTR processing with KAPAC. *Genome Biol*, **19**, 44.
12. Ye, C., Long, Y., Ji, G., Li, Q.Q. and Wu, X. (2018) APAtrap: identification and quantification of alternative polyadenylation sites from RNA-seq data. *Bioinformatics*, **34**, 1841-1849.
13. Chang, J.W., Zhang, W., Yeh, H.S., Park, M., Yao, C., Shi, Y., Kuang, R. and Yong, J. (2018) An integrative model for alternative polyadenylation, IntMAP, delineates mTOR-modulated endoplasmic reticulum stress response. *Nucleic Acids Res*, **46**, 5996-6008.
14. Cass, A.A. and Xiao, X. (2019) mountainClimber Identifies Alternative Transcription Start and Polyadenylation Sites in RNA-Seq. *Cell Syst*, **9**, 393-400.e396.
15. Harrison, B.J., Park, J.W., Gomes, C., Petruska, J.C., Sapio, M.R., Iadarola, M.J., Chariker, J.H. and Rouchka, E.C. (2019) Detection of Differentially Expressed Cleavage Site Intervals Within 3' Untranslated Regions Using CSI-UTR Reveals Regulated Interaction Motifs. *Front*

Genet, **10**, 182.
16. Wang, R. and Tian, B. (2020) APAlyzer: a bioinformatics package for analysis of alternative polyadenylation isoforms. *Bioinformatics*, **36**, 3907-3909.
17. Li, L., Huang, K.-L., Gao, Y., Cui, Y., Wang, G., Elrod, N.D., Li, Y., Chen, Y.E., Ji, P., Peng, F. *et al.* An atlas of alternative polyadenylation quantitative trait loci contri buting to complex trait and disease heritability. *Nat Genet*, **53**, 994-1005.
18. Fahmi, N.A., Ahmed, K.T., Chang, J.W., Nassereddeen, H., Fan, D., Yong, J. and Zhang, W. (2022) APA-Scan: detection and visualization of 3'-UTR alternative polyadenylation with RNA-seq and 3'-end-seq data. *BMC bioinformatics*, **23**, 396.
19. Szkop, K.J., Moss, D.S. and Nobeli, I. (2021) flexiMAP: a regression-based method for discovering differential alternative polyadenylation events in standard RNA-seq data. *Bioinformatics*, **37**, 1461-1464.
20. Gerber, S., Schratt, G. and Germain, P.L. (2021) Streamlining differential exon and 3' UTR usage with diffUTR. *BMC bioinformatics*, **22**, 189.
21. Frässle, S., Aponte, E.A., Bollmann, S., Brodersen, K.H., Do, C.T., Harrison, O.K., Harrison, S.J., Heinzle, J., Iglesias, S., Kasper, L. *et al.* (2021) TAPAS: An Open-Source Software Package for Translational Neuromodeling and Computational Psychiatry. *Front Psychiatry*, **12**, 680811.
22. Imada, E.L., Wilks, C., Langmead, B. and Marchionni, L. (2023) REPAC: analysis of alternative polyadenylation from RNA-sequencing data. *Genome Biol*, **24**, 22.
23. Long, Y., Zhang, B., Tian, S., Chan, J.J., Zhou, J., Li, Z., Li, Y., An, Z., Liao, X., Wang, Y. *et al.* (2023) Accurate transcriptome-wide identification and quantification of alternative polyadenylation from RNA-seq data with APAIQ. *Genome research*, **33**, 644-657.
24. Jonnakuti, V.S., Wagner, E.J., Maletić-Savatić, M., Liu, Z. and Yalamanchili, H.K. (2024) PolyAMiner-Bulk is a deep learning-based algorithm that decodes alternative polyadenylation dynamics from bulk RNA-seq data. *Cell reports methods*, **4**, 100707.
